# Urine‐Derived Stem Cells: Applications of Paracrine‐Driven Regeneration and Functional Recovery in Multiorgan Systems

**DOI:** 10.1155/sci/8089902

**Published:** 2026-04-27

**Authors:** Dan Li, Zhijuan Liang, Liping Wang, Yuanbin Chen, Dongkai Sun, Ye Liang

**Affiliations:** ^1^ Key Laboratory of Urology and Andrology, The Affiliated Hospital of Qingdao University, Qingdao, Shandong, China, qdu.edu.cn; ^2^ Department of Urology, The Affiliated Hospital of Qingdao University, Qingdao, Shandong, China, qdu.edu.cn

**Keywords:** paracrine effect, stem cell therapy, urine-derived stem cells, USCs-EPs

## Abstract

Stem cell therapy, a highly recognized approach in the field of regenerative medicine, is known for its potential to stimulate the self‐healing process of the body using stem cells or their derivatives, in which urine‐derived stem cells (USCs) stand out as a novel and promising tool in treating various diseases recently. USCs are widely believed to derive primarily from glomerular parietal epithelial cells and podocytes and can be readily isolated from urine. Owing to telomerase‐associated self‐renewal capacity, USCs proliferate efficiently in vitro, while their outstanding differentiation potential and paracrine effects support promising applications in tissue regeneration and functional recovery. The extracellular particles (EPs) of USCs, including various activity factors, exosomes, and other extracellular vesicles (EVs), play significant roles in the paracrine effects of USCs, which have gained recognition as pivotal factors in the therapeutic studies of USCs. USCs‐EPs can affect on diseases in different organ systems through various mechanisms, in which cell differentiation, angiogenesis, and cell proliferation and migration are the three mainly affected functions. And the USCs‐EPs may play dual roles based on different curative effects. Besides, the varied modification and encapsulation of USCs‐EPs may promote their therapeutic effects. Up to now, USCs‐EPs have been widely studied in multiorgan systems and are considered to be superior to USCs because of the rare risks of diverse genetic backgrounds, teratoma formation and immunogenicity. Although in the current preclinical stage, USCs‐EPs represent a promising novel tool in stem cell therapy and warrant further exploration for their clinical potential.

## 1. Introduction

Stem cells, a kind of unspecialized cell characterized by the ability of self‐renewal, possess the unique ability to differentiate into various cell types under specific conditions. Regenerative medicine, which revolves around stem cells and tissue engineering, has emerged as a new field in medical research. This technology aims to achieve tissue regeneration or repair by injecting stem cells or their derivatives into organisms. Stem cells can be classified into embryonic stem cells (ESCs), adult stem cells (ASCs), and induced pluripotent stem cells (iPSCs) depending on their origin, with the important roles of tissue maintenance and regeneration [1–3]. ESCs exhibit strong differentiation potential, their applications in medical treatments face challenges related to acquisition difficulties, ethical considerations, and the potential for tumorigenesis. Consequently, ASCs, including mesenchymal stem cells (MSCs), hematopoietic stem cells (HSCs), and adipose tissue‐derived stem cells (ADSCs), benefit from the easy to obtain and low risk of immunological rejection and have been partly applied to regenerative medicine. However, their limited differentiation capacity and amount of cells restrict further application. Besides, iPSCs benefit from their abundant sources are promising approaches through reprogramed cells into stem cells, used in the therapy of different diseases, while the risk of gene mutation and teratomas still needs to be resolved [4].

While ASCs have been extensively explored for stem cell‐based treatments, the extraction of many ASCs, such as bone marrow‐derived MSCs (BMSCs), involves invasive procedures that can lead to significant patient discomfort. In the past decade, a novel type of ASCs known as urine‐derived stem cells (USCs), also called urine progenitor cells or renal progenitor cells, has emerged as a promising alternative to traditional ASCs isolation procedures [5, 6]. The advantages of noninvasiveness, cost‐effectiveness, and ease of isolation make USCs an appealing option. Research has demonstrated that USCs possess robust self‐renewal capabilities and can differentiate into various lineages, including osteogenic, chondrogenic, adipogenic, and neurogenic [7, 8]. And USCs have shown outstanding vascularization potential and cell proliferation and migration activities not less than ADSCs and BMSCs, leading to excellent balanced and excellent differentiation potentials [7–10]. Therefore, even though on the preclinical stage, USCs hold great value in the realm of stem cell therapy.

Stem cell paracrine, a key characterizations of USCs, has propelled them to the forefront of regenerative medicine. Their secretome, USCs‐extracellular particles (EPs), offer a superior therapeutic paradigm by overcoming critical limitations of cell‐based approaches, such as risks of immunogenicity and tumorigenicity [11], while demonstrating consistent proregenerative functions like antisenescence and angiogenesis across diverse donors [12, 13]. However, major challenges persist. The complex, poorly defined composition of USCs‐EPs and the potential influence of donor physiology on their molecular cargo hinder standardized production and regulatory approval. Furthermore, studies on USCs‐EPs need to be further transformed from animal models to clinical trials, for which the comprehensive in vivo pharmacokinetic data and optimized delivery systems for specific organs are essential. This review synthesizes characterizations of USCs and current applications of USCs‐EPs across organ systems, providing a discussion on their clinical potential and translational challenges.

## 2. Characterizations of USCs

### 2.1. Origin of USCs

Initially, the origin of USCs was controversial due to the complex and diverse microenvironment in which they are located [14]. USCs were initially identified to express markers of stem/progenitor cells (c‐Kit and SSEA4). Further study found that USCs were positive for the basal cell marker cytokeratin 13 and especially the urothelial basal cell marker CD44 [15], suggesting to be progenitor‐like cells derived from basal cells of the urothelial layer. However, this hypothesis was challenged by studies indicating that USCs do not express other markers of uroepithelial lineage cells, including Uroplakin Ia, Uroplakin III, Zona Occludens, and epithelial cadherin [16]. Interestingly, USCs from the upper urinary tract exhibited similar characteristics with USCs from voided urine, including proliferation, potential differentiation, and chromosome stability, suggesting a potential kidney origin [16]. This viewpoint gained preliminary support when the presence of Y‐chromosomes was identified in USCs derived from a female who had received a male kidney transplant, suggesting that USCs originated from either the upper urinary tract, including the kidney, renal pelvic, and/or upper segment of the transplanted ureters [17]. And further study found that USCs expressed the markers of parietal cells and podocytes (CD146, synaptopodin, and podocin), indicating that USCs might originate from glomerular parietal epithelial cells and podocyte cells [17–19]. Lazzeri et al. [20] isolated and amplified renal progenitor cells, with the capacity to differentiate into tubular cells and podocytes, from human urine. The renal progenitor cells were also recognized as USCs, suggesting their renal origin. Furthermore, Chen et al. [21] isolated and identified two subpopulations of USCs based on distinct morphological characteristics. One spindle‐shaped group, originating from renal mesenchyme, exhibited faster proliferation and stronger osteogenic and adipogenic differentiation potential. The other rice‐shaped group showed greater capability for chondrogenic differentiation and was suggested to have a nephron tubule origin [21]. Even though many studies believed that USCs are derived from the upper urinary tract, there is no consensus on the origin of USCs. Indeed, the origin of USCs may not be homogeneous since cells in urine are complex and various. The different origin results in the heterogeneity of USCs, which may limit their application in clinical.

### 2.2. Isolation and Culture

In 2008, Zhang et al. [15] first isolated a subpopulation of progenitor‐cell‐like cells with the ability to differentiate into bladder cells from urine. Subsequently, a study in 2011 confirmed that these progenitor‐cell‐like cells possessed stem cell properties and were described as USCs [22, 23].

The donor’s urine is typically required to be fresh. Research indicated that USCs could be safely preserved in urine for up to 24 h at 4°C, maintaining high telomerase activity (TA) and bipotent differentiation capacity [24]. However, USCs might lose viability in urine without any preservation within 5 h [24–26]. Factors, such as age, gender, and the health status of urine donors, also significantly impact the extraction of USCs. It is reported that the efficiency of USC extraction was reported to be higher in males than in females [27, 28]. And cystocentesis may be a superior way of urine collection to isolating USCs [29]. Additionally, diabetes was reported to interfere with the success and quality of USC isolation, rendering them unsuitable for medical applications, while USCs from chronic kidney disease (CKD) may exhibit a stronger ability of proliferation [25, 29–31]. USCs isolated from voided urine of clear cell renal cell carcinoma (ccRCC) patients demonstrated greater proliferation and invasion capacity than those isolated from healthy donors and could express genes associated with ccRCC [32]. Similar situations were observed in patients with bladder cancer [26], suggesting the potential of USCs in predictive medicine.

The original culture medium for USCs is a mixture of keratinocyte‐serum‐free medium (KSFM) and embryonic fibroblast medium in a 1:1 ratio [15, 22]. While it has been reported that USCs can be cultured in different media [24, 27], several studies have explored various formulations of culture medium [21, 33–39]. The most commonly used medium was mainly based on the KSFM and DMEM/F12 medium with different concentrations of some specific factors, including epidermal growth factor, bovine pituitary extract, insulin, transferrin, hydrocortisone, adenine, and triiodo‐L‐thyromine. The optimal concentration of fetal bovine serum was then confirmed to be in the range of 0.5%–10% [5, 24].

Dishes precoated with collagen were found to inhibit the formation of USC colonies, as mesenchymal cells tend to adhere to plastic surfaces [27]. However, another study indicated that precoating dishes with extracellular matrix (ECM) proteins might promote the USCs proliferation after 7 days of culture; collagen‐I was identified as the most effective ECM protein [40]. This suggests that precoating might not be the determining factor for successful USCs isolation. Wang et al. [41] recently found that collagen‐I promoted USCs proliferation through the Piezo1‐ERK1/2‐YAP signaling cascade. Briefly, the mechanically activated cation channel Piezo1 was activated by the collagen‐I microenvironment, leading to calcium‐dependent activation of ERK1/2. The translocation of the transcriptional coactivator YAP to the nucleus ultimately enhances USC proliferation. Kim et al. [42] suggested that Matrigel was a superior choice for precoating compared to gelatin when extracting USCs. Moreover, the treatment with Y‐27632, an inhibitor of rho‐associated protein kinase, might greatly improve the proliferation, migration, colony formation ability, and differentiative into osteogenic or chondrogenic lineage of USCs on coated plates. The flavonoids were also considered to promote USC amplification when combined with gelatin [42].

### 2.3. Self‐Renewal Capacity

Stem cells can divide symmetrically or asymmetrically, giving rise to daughter cells that retain stem cell characteristics, that is, the process of self‐renewal. USCs can expand for at least 10 passages in vitro [15], and the capacity for self‐renewal may be associated with the age of the donors. TA has been identified as a critical factor, with a higher percentage of TA‐positive USCs found in middle‐aged donors (75%) compared to donors older than 50 (59.2%). USCs with positive TA (USCs^TA+^) exhibited enhanced proliferation, reaching up to 20 passages compared to the 8–10 passages of USCs with negative TA (USCs^TA−^) [19, 43, 44]. USCs^TA+^ also showed a stronger capacity for self‐renewal and differentiation compared to USCs^TA−^. However, the relative strength of TA in USCs^TA+^ tended to decrease with passage [44]. Besides, it is said that hypoxia may improve self‐renewal abilities of USCs by activate autophagy [45].

The clinical applications of USCs often require a substantial number of cells obtained through in vitro amplification, which is hindered by the limited passage of USCs. Therefore, strengthening the self‐renewal ability of USCs holds great significance for clinical application. In ESCs, *OCT-4* is a key gene for self‐renewal capacity [46, 47]. It is reported that USCs from different donors expressed different mRNA levels of *OCT-4* [17, 48], and it was even undetectable in some people’s USCs [49]. A recent study discovered that boldine, a small molecule compound, could activate the Wnt signaling pathway and promote the nuclear translocation of β‐catenin by inhibiting GSK‐3β. This activation ultimately led to the transcription of stemness genes, including *OCT-4* and *C-Myc* [50]. Another study aimed at enhancing the yield of USCs presented a kind of 3D silk fiber matrix, allowing long‐term culture of USCs without inducing cell senescence. This method has been successfully applied in assessing chronic mitochondrial toxicity [51].

### 2.4. Differentiation Capacity and iPSCs

USCs were described as progenitor‐like cells with the ability of self‐renewal and differentiation [15]. It is reported that age may be an influence factor for proliferation and differentiation of USCs [33, 52]. The differentiation capacity might be diverse, as shown in different studies like osteogenic, chondrogenic, adipogenic, neurogenic, and myogenic, which may attributed to the heterogeneity of USCs we mentioned above [5, 7, 9, 10, 14, 19, 48, 53]. Based on their differentiation potential, USCs have emerged as promising tools in tissue engineering and regenerative medicine, with extensive investigations into their applications for treating chronic liver disease [54–56], bone regeneration [57–59], cartilage defects [60, 61], muscular dystrophy [62, 63], acute kidney injury (AKI) [64, 65], interstitial cystitis [66, 67], urinary tract reconstruction [68–70], stress urinary incontinence (SUI) [71, 72], and neuronal regeneration [73, 74]. Several studies focused on improving the differentiation ability of USCs. Recent research has employed various approaches to improve USC differentiation, including hypoxia pretreatment, the addition of promoting factors, gene transduction, and the development of novel biomaterials [38, 62, 74–76].

IPSCs are the promising tools of regenerative medicine, by virtue of their low immunogenicity with no ethical concerns like ESCs, and can differentiate into cells of the ectoderm, mesoderm, and endoderm. It is reported that cells derived from urine, including USCs, transitional epithelial cells, urothelial cells, renal epithelial cells, and so on, can be used to generate iPSCs, which hold a superior potential for differentiation [77, 78]. Benefit from their excellent capacities of proliferative, self‐renewal, immunomodulatory, and especially differentiation [79–82], USCs have advantages over many other cells as the cell source of iPSCs. And USCs showed much more efficiency in reprogramming than MSCs by right of the epithelial origin, which may help USCs skip the mesenchymal‐to‐epithelial transition process [83, 84]. In addition to basic properties of osteogenic, chondrogenic, and adipogenic, USCs–iPSCs showed remarkable differentiated potency of skeletal muscle myocytes [85], cardiomyocytes [86–88], epithelial cells [89–91], hepatocyte‐like cells [92, 93], retinal cell [94], neurons and astrocytes [91, 95–98], and various progenitor cells [99, 100]. Therefore, USCs–iPSCs have exhibited outstanding advantages in regenerative medicine and disease modeling and drug discovery.

### 2.5. Paracrine Effect

The paracrine effect, often associated with paracrine factors, exosomes, and extracellular vesicles (EVs) represents another important characteristic of stem cells upon which stem cell therapy relies [101, 102]. The early applications of MSCs mainly focused on their differentiation potential, expecting to generate cells with entire functions to replace defective cells [48, 103, 104]. However, subsequent research showed that the paracrine effect commonly played a more important role in the therapies of certain diseases [48, 102, 105, 106]. In human, the USCs were reported to be similar to MSCs because of the similar expression of surface markers (CD73, CD90, and CD105) and common multipotent differentiation (osteogenic, chondrogenic, adipogenic, and myogenic) [9, 10, 53]. Similar to studies on MSCs, differentiation has been the focal point in the investigation of stem cell therapy with USCs. However, there has been an increasing focus on the paracrine effect of USCs. It was reported that USCs could release nonvesicular EPs (NVEPs) and EVs, which contained exosomes (Exos, usually small EVs with <200 nm in diameter) and other large EVs, to the neighboring cells and have been investigated for therapeutic applications in several diseases [14, 48].

In the stem cell therapy of USCs, the differentiation potential and the paracrine effects have become two critical research directions. USCs, especially the USCs–iPSCs based on the superior differentiation potential, have garnered significant attention due to the noninvasive and easily accessible characters, presenting a highly promising strategy for regenerative medicine. However, the clinical application still faces constraints of teratoma formation and ethical considerations. In contrast, USCs‐EPs based on the paracrine effects of USCs are gaining increasing research focus, which is largely driven by the lower risk of immunogenicity and tumorigenicity of USCs‐EPs. Given their advantages of effectiveness, convenience, and safety, USCs‐EPs were considered comparable to—and in some cases demonstrated superior efficacy to—USCs in cell therapy applications [31, 107].

**Figure 1:**
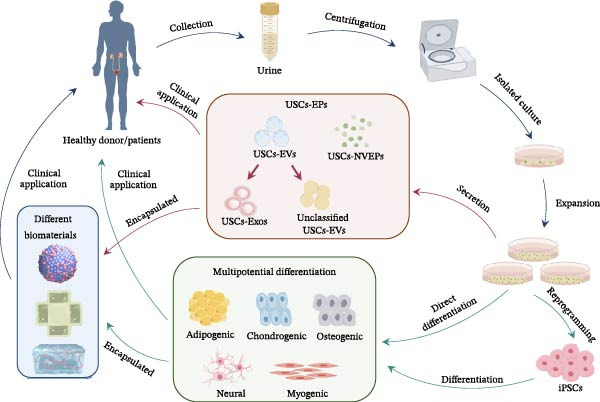
Workflow of application with USCs. The urine is obtained from healthy donors of patients and centrifugated to enrich USCs. The collected USCs are then plated in the dishes precoated with collagen‐I to isolate and further expande the culture. Collect the 2–5 passages of USCs for the next application. The application of USCs is mainly divided into two strategies based on their characteristics of multipotential differentiation and paracrine effect. And the USCs or USCs‐EPs can use directly or combined applicated with different biomaterials.

The main workflow of USCs applications is summarized in Figure 1. In brief, USCs can be obtained from the urine of patients or healthy donors. After collection, centrifugation, isolation, and amplification, USCs within 10 generations can be obtained for clinical use. USCs can differentiate into different types of cells directly or after induction into iPSCs. Meanwhile, USCs can secrete USCs‐EPs based on their paracrine characteristics. These differentiated cells based on differentiation potential and SCs‐EPs based on paracrine properties are then implanted into patients to participate in clinical therapy. Besides, it has been reported that some biomaterials can enhance the biological characteristics of USC‐differentiated cells and USCs‐EPs, which is prospective for clinical therapy [57, 76, 108–111].

## 3. Application of USCs Based on Paracrine Secretion

### 3.1. Skeletal System Diseases

#### 3.1.1. Bone Regeneration

##### 3.1.1.1. Osteoporosis

In the field of bone regeneration, USCs are considered a promising alternative to BMSCs, and their secreted EPs (USCs‐EPs) have been extensively studied in recent years. A pivotal study by Chen et al. [112] first demonstrated that systemically administered USCs‐EVs could prevent osteoporosis by targeting bone tissue and enhancing bone mass through the dual action of promoting osteogenesis and inhibiting osteoclastogenesis via CTHRC1 and OPG. Importantly, this therapeutic effect was consistent across USCs‐EVs derived from donors of varying ages, genders, and health statuses, highlighting their potential as a standardized therapeutic agent [112]. Subsequent research showing that USC‐EVs could deliver miR‐26a‐5p to osteoblast precursors. This miRNA activated the HIF‐1α/VEGFA pathway to restore osteogenic differentiation and angiogenesis by targeting HDAC4, which in turn ameliorated diabetic osteoporosis [113].

##### 3.1.1.2. Osteolysis

Periprosthetic osteolysis remains a critical challenge in joint replacement that demands effective therapeutic strategies. Li et al. [114] first established the therapeutic potential of unmodified USCs‐EVs, demonstrating their dual capacity to suppress UHMWPE‐induced osteoclastogenesis by reducing inflammatory cytokine secretion from osteoclast progenitors while concurrently promoting BMSCs osteogenic differentiation—effects robustly validated in a calvarial osteolysis mouse model. Building upon this foundation, two recent biomaterial‐based strategies have emerged to enhance USC‐EP efficacy through distinct mechanisms. Xie et al. [115] developed a macrophage membrane‐coated exosome system (MM‐Exos) designed to actively target osteolytic sites; this biomimetic platform evades macrophage phagocytosis and increases BMSCs uptake of exosomes, thereby potentiating both anti‐osteoclastogenic and pro‐osteogenic activities. In parallel, another approach utilizing PLGA nanoparticle encapsulation of USCs‐Exos has demonstrated superior efficacy in treating polyethylene‐induced osteolysis, likely through improved stability and sustained release at target sites [116]. These biomaterial‐based strategies represent significant advances in harnessing the therapeutic potential of USCs‐EPs for osteolytic diseases.

##### 3.1.1.3. Osteonecrosis and Bone Defect

Beyond their direct regulation of bone‐forming and bone‐resorbing cells, USCs‐EPs accelerate bone regeneration through complementary mechanisms involving angiogenesis and apoptosis inhibition. One study identified that USCs‐EVs carry dual functional factors of proangiogenic DMBT1 and antiapoptotic TIMP1, which collectively counteract glucocorticoid‐induced osteonecrosis. Specifically, DMBT1 preserves microvascular endothelial cell function, as evidenced by enhanced tube formation capacity, while TIMP1 suppresses apoptosis in femoral head tissues, together mitigating osteonecrosis progression [117]. Based on this mechanistic foundation, Lu et al. [108] engineered a composite hydrogel delivery system (^USC^Exos/G_5_H_2_) to prolong in vivo retention of USCs‐Exos. This bioactive scaffold not only promoted endothelial progenitor cell tube formation and upregulated angiogenic markers (CD31, FGF1, and HIF‐1α) but also enhanced osteoclastic efficacy, as demonstrated by increased trabecular number and thickness in cranial defect models. Notably, ^USC^Exos/G_5_H_2_ treatment induced abundant new bone formation with observable regenerating central canals and promoted the development of osteogenic–angiogenic H‐type vessels, providing compelling evidence for its superior dual pro‐osteogenic and proangiogenic capacity. Collectively, these studies illustrate that USCs‐EPs orchestrate bone regeneration through integrated mechanisms targeting vascular niche reconstruction and cell survival, with biomaterial‐based delivery systems amplifying these therapeutic effects.

#### 3.1.2. Cartilage Defects Repair

In 2014, it was reported that the ECM of USCs might enhance the chondrogenic differentiation capacity of BMSCs through the Wnt11 signaling pathway [39]. Recently, USCs were proved to enhance the proliferation, migration and chondrogenesis ability of theauricular‐derived chondrocyte cells by paracrine effect [118]. Likewise, a recent study showed that the USC‐derived ECM (UECM) exhibited superior promotion of chondrogenesis both in vitro and in vivo compared to ECM derived from other cells, such as synovium‐DSCs, ADSCs, and dermal fibroblasts. This superiority was attributed to the unique structure (the netlike thick fibers and iso‐directional distributive fine fibers) and expression of basement membrane proteins (higher level of COL4) in UECM, suggesting their role in enhancing the chondrogenic capacity of UECM [119, 120].

Exosomes derived from USCs are a promising tool for cartilage repair and have attracted the attention of many research teams in recent years [113, 121–124]. Tong et al. [121] identified a subpopulation of USCs as CD133^+^USCs and analyzed the function of their exosomes (USCs‐Exos and CD133^+^USCs‐Exos) in BMSCs. They found that CD133^+^USCs showed a stronger ability for chondrogenic differentiation compared to other USCs. Consequently, the CD133^+^USCs‐Exos were more effective in promoting the chondrogenic differentiation of BMSCs, while both types of exosomes demonstrated the ability to promote BMSCs’ proliferation and migration with no significant difference in vitro. In the rat model of rotator cuff injury, rats treated with complexes of CD133^+^USCs‐Exos and hydrogel exhibited thicker layers of fibrocartilage and better‐arranged fibers, as detected by H&E and TB&FG staining. Moreover, they exhibited improved biomechanical properties, highlighting the therapeutic potential of CD133^+^USCs‐Exos in rotator cuff healing. Liu et al. [122] established a rat model to investigate the function of USCs‐Exos carrying miR‐140–5p (hUSC‐140‐Exos) in knee osteoarthritis (KOA) induced by IL‐1β. They found that hUSC‐140‐Exos could enhance the proliferation and migration of cells and suppress apoptosis similar to hUSC‐Exos and downregulate the expression of VEGFA. Subsequently, this led to restoring the microstructure of subchondral bone to attenuate the abnormal angiogenesis in KOA rats. Analogously, Wan et al. [123] reported that USCs‐EVs containing miR‐26a5p, previously shown to promote osteogenic differentiation in osteoblast precursor cells [113], could promote the proliferation and migration of chondrocytes by targeting phosphatase and tensin homolog deleted on chromosome 10 (PTEN) directly with miR‐26a‐5p. This suggested the dual function of USCs‐EVs in bone and cartilage repair.

#### 3.1.3. Intervertebral Discs (IVD) Protection

Intervertebral disc degeneration (IDD) may result in various common diseases, such as lower back pain, with the principal pathogenesis involving the degeneration of the nucleus pulposus (NP) [125]. Recently, the USCs‐Exos was found beneficial to IDD. It is reported that NP cells under stress experienced endoplasmic reticulum (ER) stress and apoptosis, which could be inhibited by incubating with USCs‐Exos. It was demonstrated that USCs‐Exos reduced the ER stress‐induced unfolded protein response and suppressed apoptosis through the AKT/ERK pathway in NP cells, thereby alleviating IDD [126, 127]. The reduction of NP cells and ECM could also contribute to IDD [128]. Guo et al. [129] treated NP cells obtained from IDD patients with USCs‐Exos in vitro and found salutary changes in NP cells, such as enhanced proliferation, increased ECM synthesis (especially the COL2 and ACAN), and resistance to senescence. MATN3, a protein downregulated significantly in IDD rats, was the major activity factor and enriched in USCs‐Exos. Knockdown of MATN3 in USCs‐Exos weakened the salutary changes in NP cells caused by USCs‐Exos through TGF‐β/Smad and PI3K/Akt signaling pathways. Furthermore, USCs‐Exos could alleviate IVD degeneration, resulting in higher disc height, increased ECM synthesis, and well‐organized NP tissues in IVD rats. These functions were weakened upon inhibition of the exosomal MATN3.

**Figure 2:**
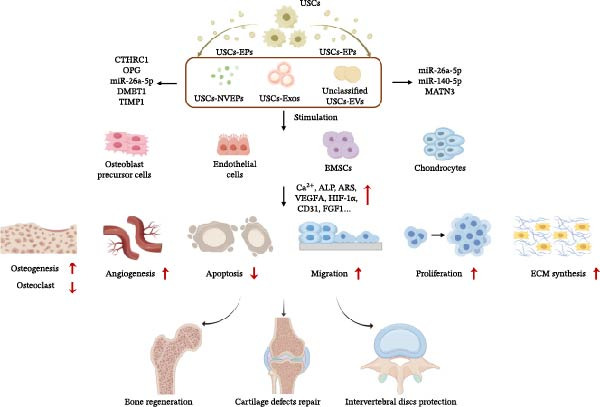
The main mechanisms of USCs‐EPs in the application of skeletal system diseases. The mainly treated cells are osteoblast precursor cells, endothelial cells, BMSCs and chondrocytes. The USCs‐EPs can work through promoting cell proliferation, migration, angiogenesis, ECM synthesis and osteogenesis differentiation, as well as inhibiting apoptosis and osteoclast differentiation to repair the defects of bone and cartilage.

In summary, diseases of the skeletal system are one of the main application areas of USCs, consistently occupying a prominent position in USC research. USCs‐EPs, a promising tool for free‐cell treatment, have gained considerable attention and have been widely investigated in recent years, which suggested a promising therapeutic direction involving USCs‐EPs. The USCs‐EPs might play various roles in clinical applications of skeletal system diseases. The primary therapeutic mechanisms from key studies in this context are summarized in Figure 2. USCs‐EPs can stimulate osteoblast precursor cells, endothelial cells, BMSCs, and chondrocytes to promote osteogenesis, angiogenesis, cell proliferation and migration, and ECM synthesis, while simultaneously inhibiting apoptosis and osteoclastogenesis. And finally contributes to enhanced bone regeneration and cartilage repair.

### 3.2. Genitourinary System Diseases

#### 3.2.1. Nephropathy

##### 3.2.1.1. Diabetic Nephropathy (DN)

USCs are an appropriate cell source of nephropathy due to their robust renal lineage differentiation, immunomodulatory effects, high secretion of growth factors, and paracrine effects [130]. DN, the main complication of diabetes, is early marked by damaged glomerular podocytes induced by hyperglycemia [131]. Studies reported that USCs‐Exos contained various factors and microRNAs, which targeted the key proteins associated with angiogenesis and cell survival, like VEGFA, could prevent podocyte and tubular epithelial cells from apoptosis caused by high glucose levels and promoted the proliferation of glomerular endothelial cells both in vivo and in vitro, subsequently preventing kidney injury in diabetes [132, 133]. Besides, USCs‐Exos were also reported to inhibit autophagy of kidney cells through the mTOR pathway, as well as promoted macrophage M2 polarization through the SOCS1/STAT3 axis, to inhibit DN progression [134, 135]. These findings underscore the significance of targeting vessel regeneration and podocyte protection in the therapeutic effects of USCs‐Exos on DN.

##### 3.2.1.2. AKI

AKI, with high morbidity and mortality rates, is a common syndrome in clinical. It is reported that USCs exhibited robust protective effects on tubular cells and anti‐inflammatory effects on macrophages in vitro and released various cytokines and growth factors to promote proliferation as well as inhibit apoptosis of tubular cells in AKI mice [136–138]. Ischemia‐reperfusion injury (IRI) is believed to be the major cause of AKI. In vitro and in vivo studies indicate that USCs and their derived EPs can mitigate tubular cell injury, suppress apoptosis, and modulate inflammatory responses, particularly in IRI models [131, 139, 140]. Notably, their reparative potential extends beyond the kidney, as evidenced by their ability to promote muscle regeneration and angiogenesis in murine hindlimb ischemia models [141]. Mechanistically, USCs‐EVs such as miR‐146a‐5p, miR‐122‐5p, and miR‐216a‐5p, play a crucial role by targeting key signaling pathways (e.g., IRAK1/NF‐κB and SOX2, PTEN) to reduce inflammation, fibrosis, and cell death in IRI‐AKI rats [138, 142, 143]. Additionally, targeting circDENND4C with USCs‐Exos has shown potential in attenuating pyroptosis, suggesting another therapeutic avenue [144, 145].

##### 3.2.1.3. CKD

Apart from exosomes, paracrine factors have also been investigated in several renal diseases. It was found that Klotho could suppress TGF‐β in HK2, potentially inhibiting fibrosis and promoting the function of HK2, thereby making it a key therapeutic factor for CKD [145, 146].

As kidney‐derived cells, USCs have natural advantages in the treatment of nephropathy compared to MSCs, HSCs, ADSCs, and so on. Further in‐depth studies might help in understanding the role and mechanism of USCs and their paracrine effect in the treatment of nephropathy, thereby aiding in their clinical applications.

#### 3.2.2. Bladder and Urinary Tract Diseases

##### 3.2.2.1. Bladder Outlet Obstruction (BOO)

BOO, a common urinary system disease, is characterized by increased bladder outlet resistance pressure and intravesical pressure, often leading to inflammatory response and fibrosis of bladder tissues [147, 148]. In 2022, USCs were first used to treat BOO, demonstrating their efficacy in bladder remodeling and functional protection in rats with partial BOO. The analysis of the miRNA‐gene interaction network showed that the protective effect was attributed to two miRNAs, including miR‐142 and miR‐9a, while the underlying mechanism was not illustrated [148]. In the same year, Wu et al. [149] administered USCs‐Exos into the bladders of BOO mice, observing significant reductions in the expression of fibrotic markers α‐SMA and collagen III, along with attenuation of bladder fibrosis. Bioinformatics analysis identified nuclear respiratory factor 1 (NRF1) as a key mediator of this anti‐fibrotic effect. In vitro, TGF‐β1‐induced bladder smooth muscle cells exhibited elevated levels of TGF‐βR1, α‐SMA, and collagen III, all of which were reversed by USCs‐Exos treatment. This therapeutic effect was abolished upon knockdown of NRF1 in USCs‐Exos. Additionally, miR‐301b‐3p was identified as the downstream target of NRF1, through which it inhibits TGF‐βR1 activation and prevents fibrosis progression.

##### 3.2.2.2. SUI

Pelvic floor muscle especially the pubococcygeus muscle, injury is the primary pathogenesis of SUI [150, 151]. Consequently, USCs, known for their robust myogenic differentiation potential, are considered a promising cell source for treating SUI. Additionally, the paracrine effects of stem cells recognized as another potential mechanism [70, 152] also suggested the potential value of USCs in SUI treatment. Early in 2013, USCs proved to be helpful to SUI with identified VEGF as the potential functional factor. The overexpression of VEGF in USCs enhanced the angiogenesis, cell proliferation, and myogenic differentiation of the implanted cells and promoted innervation [72]. Wu et al. [153] treated SUI rats with USCs‐Exos and found a significant promotion of urodynamic parameters. Notably, USCs‐Exos demonstrated the ability to reduce muscle fibrosis, improve muscle morphological recovery, and repair the damage to the contraction ability of the injured pubococcygeus muscle in SUI rats. Besides, USCs‐Exos could activate satellite cells by promoting the Ras‐ERK signaling pathway, stimulating muscle regeneration.

#### 3.2.3. Genital Diseases

##### 3.2.3.1. Erectile Dysfunction (ED)

ED is a serious genital disease that imposes significant stress and mental burden on patients, adversely affecting the overall quality of life for entire families. The paracrine effect of USCs has emerged as a novel therapeutic strategy for ED. Given the diverse contributing factors to ED, the therapeutic mechanisms of USCs may vary depending on the specific pathogenesis of ED. Diabetes is a common pathogenesis of ED. In 2014, Ouyang et al. [154] treated diabetic ED (DED) rats with FGF2 secreted by USCs. They found notable high endothelial expressions of CD31, VEGF, and endothelial nitric oxide synthase, as well as an increased proportion of smooth muscle cells. Consequently, the mean arterial pressure and intracavernosal pressure were significantly improved [154]. It is also reported that USCs‐EVs have a powerful angiogenic function, providing the possibility for the regeneration of the corpus cavernosum [155]. Subsequent studies confirmed the efficacy of USCs‐EPs in alleviating DED [109, 156–158]. Zhuang et al. [109] demonstrated that local administration of hyaluronic acid‐loaded USC‐EVs (USC‐EVs‐HA) into DED rats promoted erectile function recovery by reducing corpus cavernosum apoptosis and fibrosis. This treatment enhanced endothelial cell function both in vitro and in vivo, with prolonged application yielding superior therapeutic outcomes. Meanwhile, Zhang et al. [158] elucidated a distinct autophagy‐related pathway. Using AGEs to induce diabetic‐like injury in cavernosal endothelial cells in vitro, they found that co‐culture with USCs restored autophagic function and protected cells from damage. In vivo studies confirmed these effects, showing that USC injection repaired cavernosal endothelial autophagy in DED rats, which was evidenced by elevated LC3‐II/LC3‐I and Beclin1 levels alongside reduced p62 expression, thereby restoring endothelial and erectile function. Collectively, these findings highlight that USCs alleviate diabetic ED through multiple paracrine mechanisms, targeting both structural integrity through EV‐mediated anti‐fibrotic effects and cellular homeostasis via autophagy restoration.

Beyond diabetes, numerous other diseases might contribute to ED. ED always occurs after radical prostatectomy of prostate cancer, primarily due to neuropraxia, fibrosis, and hypoxia [159]. In a study addressing neurogenic ED following bilateral cavernous nerve injury, Yang et al. [160] demonstrated that pigment epithelium‐derived factor secreted by USCs protected penile dorsal nerves—evidenced by increased ratios of nNOS‐, nestin‐, and NF200‐positive fibers—while concurrently suppressing apoptosis, enhancing endothelial content, and improving the smooth muscle‐to‐collagen ratio in cavernous tissue, collectively restoring erectile function. In contrast, Peyronie’s disease (PD) involves localized fibrotic plaque formation. Research shows that USC‐derived exosomes can ameliorate ED in PD models through a dual antifibrotic mechanism: they inhibit myofibroblast formation in the tunica albuginea via suppression of the TGF‐β1/Smad pathway while simultaneously modulating ECM remodeling by upregulating MMPs and downregulating TIMPs, thereby reducing collagen deposition [161]. Together, these studies illustrate the multifaceted therapeutic potential of USCs and their paracrine factors in ED of distinct origins, exerting benefits through nerve protection, antiapoptotic, proangiogenic, and multitargeted anti‐fibrotic mechanisms.

##### 3.2.3.2. Nonobstructive Azoospermia (NOA)

The paracrine effects of USCs also extend to other reproductive tract disorders, including NOA and postsurgical vaginal injury [110, 162]. In busulfan‐induced NOA mice, administration of USCs‐Exos enhanced the expression of spermatogenic genes and proteins, increased testicular and epididymal weight ratios, and thickened the seminiferous epithelium after 30 days, indicating a potential role in restoring endogenous spermatogenesis. Notably, however, USCs‐Exos did not provide early protection against busulfan‐induced damage, as no spermatogenesis was observed within the first 3 days of treatment [162]. In a model of vaginal injury repair, Xu et al. [110] delivered USCs‐EVs encapsulated in a photo‐triggered imine crosslinked hydrogel (piGEL). This piGEL‐EVs system promoted vaginal mucosal wound healing in rabbits by enhancing reepithelization and angiogenesis. Mechanistically, miR‐126‐3p within USCs‐EVs was shown to facilitate vaginal epithelial cell migration and differentiation via the ERK1/2 and AKT pathways, thereby accelerating mucosal repair.

**Figure 3:**
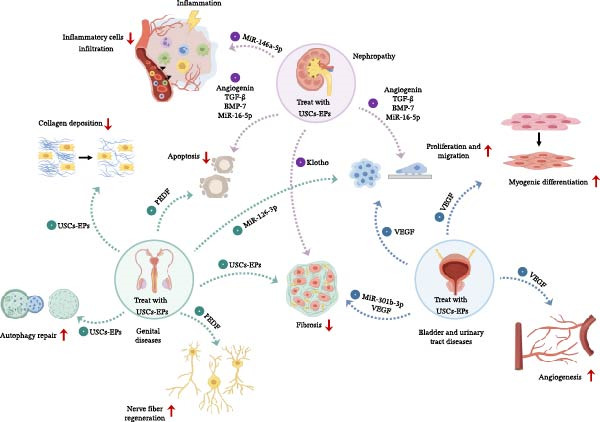
The main mechanisms of USCs‐EPs in the application of genitourinary system diseases. The main mechanisms of nephropathy are promoting cell proliferation, migration and inhibiting apoptosis, cell fibrosis, and inflammatory cell infiltration. The mechanisms of bladder and urinary tract diseases are promoting cell proliferation, migration, angiogenesis, and myogenic differentiation, as well as suppressing cell fibrosis. The main mechanisms of genital diseases are improving cell proliferation, migration, nerve fiber regeneration and autophagy repair, as well as inhibiting apoptosis, cell fibrosis and collagen deposition.

In conclusion, USCs‐EPs are a promising therapeutic tool for urogenital diseases, which can play different roles in various diseases. USCs‐EPs facilitate urological tissue repair by stimulating resident urinary system cells and modulating key biological processes, such as collagen deposition, apoptosis, cell differentiation, angiogenesis, tissue fibrosis, and autophagy. It can be found that promoting cell proliferation and migration, inhibiting cell apoptosis, and inhibiting fibrosis might be the most common mechanisms of USCs‐EPs in treating different urogenital diseases (Figure 3).

### 3.3. Other Diseases

#### 3.3.1. Skin Wound Healing

##### 3.3.1.1. General Skin Wound

Stem cells have found widespread applications in skin wound repair due to their remarkable differentiation potential and diverse effects of paracrine signaling. In 2014, USCs were shown to secrete VEGF and TGF‐β1, contributing to the promotion of wound healing. Fu et al. [36] seeded USCs onto polycaprolactone/gelatin (PCL/GT) membranes, known for their biocompatibility, to synthesize USCs‐PCL/GT, which were used to treat wounds in rabbits. After 14 days of treatment, it was found that USCs‐PCL/GT could increase the microvessel density of the wound, accelerating wound healing. In vitro studies showed that conditioned medium (USCs‐CM), rich in VEGF and TGF‐β1, could enhance proliferation, migration, and tube formation of human umbilical vein endothelial cells (HUVECs). This suggested that the paracrine effect of USCs promoted the angiogenesis of HUVECs, facilitating wound healing. In addition to promoting angiogenesis, USCs have been confirmed to improve collagen deposition by paracrine effects, further contributing to improved wound healing. Zhang et al. [163] reported a kind of bio‐glass combined with USCs‐CM, demonstrating enhanced paracrine interactions between USCs and HUVECs, resulting in improved angiogenesis and collagen deposition. This presented a novel avenue for exploring the possibility of wound healing. Interestingly, studies have reported that USCs‐Exos contribute to the suppression of collagen deposition in PD‐induced ED to inhibit cell fibrosis [161]. It seems that USCs‐EPs play a dual role in collagen deposition. When damaged tissues require repair, USCs‐EPs can promote collagen deposition to accelerate tissue repair; when collagen accumulates excessively and results in fibrosis, USCs‐EPs may inhibit collagen deposition to suppress cell fibrosis.

##### 3.3.1.2. Chronic Nonhealing Ulcer

The chronic nonhealing ulcer is a common complication in diabetes, with severe cases potentially leading to amputation. Chen et al. [164] conducted proteomic analysis and found that DMBT1 was enriched in USCs‐Exos. DMBT1, known for promoting angiogenesis, could enhance the angiogenic ability of endothelial cells. Studies in vivo confirmed the proangiogenic effect of exosomal DMBT1. Treatment with USCs‐Exos could improve angiogenesis and facilitate wound repair in diabetic mice, and the effect could be neutralized by inhibiting the expression of DMBT1. Recently, Wang et al. [165] developed a kind of bioactive patch which combined ECM of USCs with porcine small intestinal submucosa (SIS). The patch was applied to treat diabetic ulcers in type I diabetic rats, achieving remarkable outcomes through the paracrine effect of USCs. The effective factor was then confirmed to be miR‐486‐5p which targeted SERPINE1 of endothelial cells, and might facilitate healing of diabetic wounds through enhanced proliferation and angiogenesis [166].

#### 3.3.2. Nervous System Injury

##### 3.3.2.1. Ischemic Neuropathy

USCs exhibit multiple effects in providing protection to the nervous system and facilitating damage repair. It is reported that USCs can improve neural function of cerebral ischemia‐reperfusion based on the paracrine effect [167]. In a study involving ischemic stroke rats, the administration of USCs‐Exos yielded significant outcomes, such as reduced infarct volume, enhanced functional recovery, and increased neurogenesis. Moreover, neural stem cells were induced by oxygen‐glucose deprivation/reoxygenation to mimic ischemic stroke in vitro, and neural stem cells treated with USCs‐Exos showed increased cell proliferation and neuronal differentiation. Further evidence showed that USCs‐Exos carrying miR‐26a could inhibit the expression of histone deacetylase 6 and mediate the promotion of neuronal differentiation [168]. Pan et al. [169] conducted a study using a rat model with neurological deficits induced by cardiopulmonary resuscitation (CPR) following cardiac arrest (CA). The transplantation of USCs into CA/CPR rats demonstrated the protective effects of USCs on neurological function. The transplanted USCs secreted significant amounts of brain‐derived neurotrophic factor (BDNF) and VEGF, acting on the hippocampus and temporal cortex to recover the injured brain tissue and improve neurological function.

##### 3.3.2.2. Rett Syndrome (RTT)

RTT is a rare progressive neurodevelopmental disorder characterized by early neurologic dysfunction [170]. Pan et al. [171] showed that miR‐21‐5p derived from USCs‐Exos targeted Eph receptor A4 (EphA4) and regulated the TEK to promote the differentiation of NSCs. It was confirmed that miR‐21‐5p enhanced the expression levels of doublecortin and β‐III tubulin, as well as increased the content of neuron class III beta‐tubulin‐positive and doublecortin‐positive nerve cells both in vivo and in vitro. The exosomal miR‐21‐5p could target the NSCs in the subventricular zone of the lateral ventricle to improve neuronal differentiation and further promote the behavioral, cognitive, and motor coordination abilities of RTT mice.

#### 3.3.3. Oral Diseases

##### 3.3.3.1. Periodontal Diseases

Periodontal diseases, including gingivitis and periodontitis, often lead to the destruction of periodontal tissues. Human periodontal ligament stem cells (hPDLSCs) are considered ideal seed cells of periodontal defect regeneration, and USCs have been reported to promote the repair function of hPDLSCs [172, 173]. Yang et al. [172] cocultured USCs with hPDLSCs and found large secretions of VEGF and basic fibroblast growth factor (b‐FGF) in a conditioned medium, which could promote the proliferation and osteogenic differentiation of hPDLSCs both in vitro and in vivo. The appropriate ratio of hPDLSCs to USCs was determined to be 1:2, resulting in the regeneration of denser collagenous fibers. Xiong et al. [173] cultured USCs onto the tissue culture plastic enriched with fibronectin to produce UECM, generating dense bundles of fibers. The subsequent seeding of hPDLSCs onto UECM demonstrated a more robust promotional effect on the proliferation and differentiation of hPDLSCs compared to seeding on ECM derived from hPDLSCs (PECM). Moreover, hPDLSCs cultured with UECM exhibited superior potential for osteogenic, adipogenic, and angiogenic differentiation. Additionally, the promotion effect of UECM on hPDLSCs was stronger than fibronectin even though fibronectin was the main component of UECM.

##### 3.3.3.2. Salivary Glands Hypofunction

Hypofunction and radiation‐induced xerostomia of salivary glands are predominant complications of head and neck cancer following radiotherapy. In a recent study, SG rats were subjected to ionizing radiation to induce injury and subsequently treated with USCs‐Exos. It was found that USCs‐Exos could decrease expression levels of a‐SMA and upregulate stem cell growth factor receptor (c‐Kit) through the Wnt3a/GSK3β signaling pathway. Additionally, exosomes from hypoxia‐pretreated USCs might have a stronger ability to repair IR‐induced injury of salivary glands [174].

#### 3.3.4. Inflammatory and Immune Diseases

It was reported that USCs secreted various immunomodulatory cytokines and possessed great potential in treating immunopathy [53, 79]. Wu et al. [79] cocultured peripheral blood mononuclear cells with USCs and found that USCs secreted more immunomodulatory cytokines, including IL‐6, IL‐8, monocyte chemoattractant protein 1, RANTES, recombinant human growth regulated protein (GROα), and granulocyte‐macrophage colony‐stimulating factor (GMCSF) compared to BMSCs. Furthermore, the cytotoxicity of natural killer cells toward USCs was much lower than that observed for BMSCs and human smooth muscle cells. The exceptional immunomodulatory properties of USCs are attributed to their robust paracrine effect, which is considered comparable to that of BMSCs. Furthermore, USCs‐EVs were found to possess immunomodulatory functions, containing cytokines, such as IL‐6, CD40L, B‐cell‐activating factor (BAFF), and a proliferation‐inducing ligand (APRIL), and could enhance the proliferation and IgM secretion of B cells. This suggested that USCs‐EVs could serve as a novel cell‐free approach for immunodeficiency therapy by activating B cell immune response [175].

Inflammatory bowel diseases (IBDs) usually result from dysfunction of T cells, particularly Th1 and Th17 CD4^+^ T cells. It was found that USC treatment could alleviate and even protect against acute colitis, as evidenced by a longer colon length, decreased infiltration of inflammatory cells, fewer epithelial ulcerations, relieved intestine inflammation, and improved overall survival of colitis mice. Moreover, prostaglandin E2 (PGE2), secreted by USCs, was proved to be responsible for the improvement of colitis mice by reducing Th1/Th17 inflammation response [80]. Besides, USCs secreted high levels of immunomodulation cytokines like IL‐6, IL‐8, MCP‐1, and so on in the microenvironment of rheumatoid arthritis, which enhanced the immunomodulatory function of USCs. The activated PBMCs significantly were then inhibited by USCs significantly, indicating that USCs intraarticular injection might be an efficiency therapeutic in the rheumatoid arthritis treatment [176].

#### 3.3.5. Ophthalmic Diseases

The USCs were also reported to have an effect on retinal cells and to participate in the repair of retinal injuries through paracrine signaling. Shi et al. [177] treated aging retinal pigment epithelial (RPE) cells induced by D‐galactose with USCs‐CM and found a significant improvement in cell morphology. The treatment with USCs‐CM led to a reduction in the content and apoptosis rate of aging RPE cells. Bioinformatic analysis revealed that after culturing with USCs‐CM, 90 genes were upregulated and 75 genes were downregulated in the aging RPE cells, in which top three enriched signaling pathways were cytokine–cytokine receptor interaction, IL‐17 signaling pathway and ferroptosis. Similarly, USCs‐CM could promote the viability and proliferation of aging retinal ganglion cells. The subsequent analysis of differentially expressed genes showed that 137 genes were upregulated, and 517 genes were downregulated in aging retinal ganglion cells. And the top three enriched signaling pathways were cytokine–cytokine receptor interaction, Jak‐STAT signaling pathway, and circadian entrainment [178]. It seemed that cytokine–cytokine receptor interaction play most important roles in USCs‐CM treatment of aging RPE cells.

**Figure 4:**
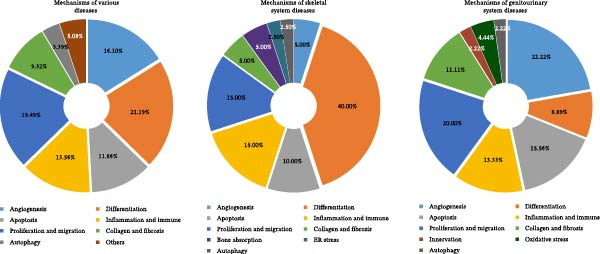
The pie chart of USCs‐EPs treating mechanisms in the skeletal system, genitourinary system and all diseases. In total 60 researches are brought into statistics, and the proportion of each mechanism is present on the pie chart.

In summary, the studies on USCs‐EPs used for the treatment of different diseases are listed in Table 1, and the proportion of major treatment mechanisms in disease researches are summarized in Figure 4. It was found that among all studies on the therapeutic application of USCs‐EPs, cell differentiation (21.19%), angiogenesis (16.1%), and proliferation and migration (19.49%) are the main mechanisms affected by USCs‐EPs and are widely researched. In skeletal system diseases, regulation of cell differentiation, including promoting osteogenic differentiation and inhibiting osteoclastic differentiation, is the focus of the treatment. In genitourinary system diseases, promoting angiogenesis and endothelial cell function has become the core therapeutic mechanism. Interestingly, USCs‐EPs often play different roles in treating different diseases. As we mentioned above, the effects of USCs‐EPs on collagen deposition differently depend on the therapeutic purpose of inhibiting fibrosis or promoting wound healing. However, compared to the abundant types and effects of USCs‐EPs, the current studies are far from covering their mechanisms in the treatments of various diseases. Therefore, further research is still needed to reveal their therapeutic mechanism.

**Table 1:** Applications of USCs‐DVs in various diseases.

Application area		USCs‐EPs	Effected factor	Function	Targeted	Biomaterial	Disease	Ref
Skeletal system	Bone	Unclassified USCs‐EVs	CTHRC1, OPG, miR‐26a‐5p, DMBT1, TIMP1	Activate osteogenesis; Inhibit osteoclast; decrease inflammation; promote angiogenesis; suppress apoptosis	HDAC4/HIF‐1α/VEGFA axis	—	Osteolysis, Osteoporosis	[112–114, 117]
		USCs‐Exos	—	Activate osteogenesis; promote angiogenesis; Inhibit osteoclast; decrease inflammation; reduced bone absorption	—	GelMA‐HAMA/Nhap; PLGA‐NPs; macrophage membrane encapsulated	Osteonecrosis, Osteolysis	[108, 115, 116]
	Cartilage	Unclassified USCs‐EVs	miR‐26a‐5p	Promote cell proliferation and migration	PTEN	—	Cartilage defects, KOA	[123]
		USCs‐Exos	MATN3, miR‐140‐5p, cd133	Promote proliferation and migration; promote ECM synthesis; Suppress apoptosis; Inhibit ER stress; promote chondrogenic differentiation; Inhibit osteoclast; decrease inflammation	TGF‐β/SMAD/AKT; AKT/ERK; VEGFA	Hydrogel	IDD, KOA, Temporomandibular joint osteoarthritis Rotator cuff injury	[121, 122, 126, 127, 129]
		Unidentified	—	Promote cell proliferation; promote chondrogenesis; promote chondrogenic differentiation; modulated local immune responses; Inhibit autophagy	wnt11 pathway	Small intestinal submucosa	Cartilage defects	[39, 118–120]
Genitou‐rinary System	Kidney	Unclassified USCs‐EVs	—	Decrease inflammation; reduce circulating stress response; promote cell proliferation; promote angiogenesis	—	—	Renal IRI	[140, 141]
		USCs‐Exos	miR‐146a‐5p, miR‐216a‐5p, miR‐16‐5p, Angiogenin, TGF‐β, BMP‐7, ATG7, circ DENND4C	Inhibit oxidative stress; decrease inflammation; suppress apoptosis; promote angiogenesis; promote cell proliferation; inhibit autophagy; inhibits M1 macrophage polarization	IRAK1/NF‐κB, PTEN/AKT, VEGFA, mTOR, circ DENND4C/miR 138‐5p/FOXO3a, SOCS1/STAT3	—	Renal IRI, AKI, DN	[132–135, 138, 142–144]
		USCs‐NVEPs	Klotho	Inhibit cell fibrosis	TGF‐β	—	CKD	[145, 146]
		Unidentified	EGF	Decrease inflammation; Inhibits macrophage polarization; promote cell proliferation; suppress apoptosis	—	—	AKI	[136, 137]
	Bladder/urinary tract	USCs‐Exos	NRF1	Promote cell proliferation and migration; inhibit cell fibrosis; Inhibit cell fibrosis; stimulate muscle regeneration	Ras/ERK, miR‐301b‐3p/TGF‐βR1	—	SUI, BOO	[149, 153]
		USCs‐NVEPs	VEGF	Promote angiogenesis; promote myogenic differentiation; promoted innervation; promote cell proliferation and migration	—	Collagen‐I gel	SUI	[72]
	Genitals	Unclassified USCs‐EVs	miRNA (miR‐21‐5p, let‐7 family, etc.), miR‐126‐3p	Suppress apoptosis; promote angiogenesis; promote cell proliferation; enhance antioxidant capacity; promote epithelization	Spred1/PIK3R2/ ERK/ATK	HA; crosslink hydrogels; void‐forming photoinduced imine crosslinking hydrogel	DED, Vaginal injuries, Corpus spongiosum defect	[109, 110, 155, 156]
		USCs‐Exos	—	Inhibit cell fibrosis; reduced the collagen deposition; promote myogenic differentiation; promote seminiferous epithelium	TIMPs/MMPs	—	PD, NOA	[161, 162]
		USCs‐NVEPs	FGF2, PEDF	Promote endothelial expressions; promote angiogenesis; Inhibit cell fibrosis; suppress apoptosis; increase smooth muscle content and reduce collagen deposition	—	—	DED, CNIED	[154, 160]
		Unidentified	—	Reduce collagen deposition; suppress apoptosis	—	—	DED	[157, 158]
Skin		USCs‐Exos	DMBT1	Promote angiogenesis; promote cell proliferation and migration	—	—	Diabetic ulcers	[164]
		USCs‐NVEPs	VEGF, TGF‐β1, b‐FGF	Promote tube formation; stimulate reepithelialization; promote angiogenesis; promote cell proliferation and migration; promote collagen formation	—	Polycaprolactone/gelatin; bioglass	Wound healing	[36, 163]
		Unidentified	miR‐486‐5p	Promote angiogenesis; promote collagen deposition; stimulate reepithelialization; modulate inflammatory response; promote cell proliferation and migration;	SERPINE1	Bioactive patch of porcine small intestinal submucosa; dECM hydrogel	Diabetic ulcers	[165, 166]
Nervous system		USCs‐Exos	miR‐26a, miR‐21‐5p	Promote cell proliferation; promote neuronal differentiation	HDAC6, EPha4/TEK	—	Ischemic stroke, RTT	[168, 171]
		USCs‐NVEPs	BDNF, VEGF	Suppress apoptosis	—	—	Neurological deficits	[169]
Oral		USCs‐Exos	—	Inhibit cell fibrosis; activate differentiation of stem cell	Wnt3a/GSK3β signaling pathway	—	IR‐induced injury of SGs	[174]
		USCs‐NVEPs	VEGF, b‐FGF	Promote osteogenic differentiation; promote cell proliferation	—	—	Periodontal diseases	[172]
		Unidentified	—	Promote osteogenic, adipogenic, and angiogenic differentiation; promote angiogenesis; promote cell proliferation, attachment and spreading	—	—	Periodontal diseases	[173]
Inflammatory and immune		Unclassified USCs‐EVs	IL‐6, BAFF, CD40L, APRIL	Enhance the proliferation and IgM secretion of B cells; activate B cell immune response	—	—	—	[175]
		USCs‐NVEPs	IL‐6, IL‐8, MCP‐1, RANTES, GROα, GMCSF, PGE2	Activate T cell immune response; inhibit proliferation of activated PBMCs	Th1/Th17 inflammation response	—	IBD	[80, 176]
Ophthalmology		Unidentified	—	Suppress apoptosis; promote cell proliferation	—	—	Retinal injury	[177, 178]

### 3.4. Opportunities and Challenges

USCs are recognized as an excellent alternative to MSCs due to their several beneficial properties, including multidifferentiation, noninvasive, safe, and cost‐effective acquisition, as well as significant potential in regenerative medicine. However, some limitations hinder their widespread applications. First, the susceptibility of USCs to microbial contamination is a concern, particularly in the context of urine collection, especially in women and children [179]. Second, USCs have the potential to extend up to 60–70 generations, sufficient for clinical treatment [17, 24, 180]; however massive in vitro amplification is challenging. Third, different markers, gene expression, and cell morphology have been reported in studies, which may result from the various isolation and culture conditions, even the diverse genetic backgrounds [14, 28, 31]. Fourth, different genetic backgrounds have been proven to impact the properties of USCs‐derived iPSCs, raising concerns about clinical issues, such as teratomas [14, 181]. Additionally, the immunogenicity of allogeneic USCs requires careful consideration. Thus, there is a need for the establishment of further quality control measures and safety standards for the application of USCs.

Since there is some indeterminacy in using USCs directly for clinical treatment, the advantages of USCs‐EPs have emerged gradually. Distinguishing whether the therapeutic effects of USCs are attributed to their multidifferentiation potential or paracrine effect in vivo is challenging, partially due to difficulties in tracing the labeled USCs within repaired tissues [5]. Notably, markers detected in the target cells of stem cell therapy are believed to originate mainly from the transfer of cell‐surface markers by stem cell derivatives rather than cell fusion [182–184], emphasizing the pivotal role of paracrine signaling in the protective effect of USCs on damaged tissues. Moreover, treatment with USCs‐EPs might remove several restrictions on the application of USCs. Obtaining abundant USCs‐EPs for in vitro treatment is more feasible with lower economic and time costs. Furthermore, the use of allogeneic USCs‐EPs presented fewer ethical concerns and reduced risks associated with diverse genetic backgrounds, teratoma formation, and immunogenicity compared to the use of USCs or USCs–iPSCs directly. As previously discussed, the heterogeneity in the cellular origin of USCs may constrain their clinical application. In contrast, the USCs‐EPs present a promising strategy to mitigate this limitation. Evidence indicates that USCs‐EPs can effectively inhibit cellular senescence, and this bioactivity appears largely independent of donor variables such as age, gender, or health status [12, 13]. This donor‐independent functional profile suggests that USCs‐EPs could offer a more consistent therapeutic effect, thereby broadening their potential for clinical translation. While current evidence confirms their analogous bioactivity, it is acknowledged that the specific molecular composition of USCs‐EPs may still correlate with the donor’s physiological state. Therefore, further characterization of the relationship between donor profiles and the composition of USCs‐EPs will be valuable for fully realizing their clinical potential.

While the clinical applications of stem cell derivatives, particularly USCs‐EPs, are in their early stages, they have encountered numerous challenges. The optimization of separation and purification methods, especially for clinical derivatives, such as exosomes, is crucial and requires the establishment of comprehensive quality standards. And the USCs‐EPs isolation should be more effective. Recent studies reported that controlled release of hepatic growth factor can significantly promote the secretion of small EVs of USCs, which provide a new pathway to increase the yield of USCs‐EPs [185]. Moreover, due to the short retention time of most derivatives in peripheral blood, the development of an efficient delivery system targeting damaged tissues is essential. In‐depth studies on USCs‐EPs are currently limited; most studies focus on verifying their effects in animal models, leaving the specific mechanisms requiring further exploration. It has been reported that USCs extracted from patients with bladder or kidney cancer are highly invasive and express related genes [26, 32]; however, the characteristics of their derivatives have not been verified, posing elevated requirements for the use of autologous USCs. Furthermore, while some studies on exosomes have been carried out in animals and even clinical trials, experimental studies on USCs‐EPs are still limited to small model organisms.

Apart from the applications mentioned above, USCs and their derivatives hold significant promise for advancing personalized and predictive medicine. As we mentioned above, as an autologous cell source obtainable noninvasively, USCs circumvent immune rejection and are prime candidates for patient‐specific regenerative therapies [186, 187]. Furthermore, USCs serve as promising tools for disease modeling, since they can be reprogrammed into patient‐specific iPSCs to construct genetically accurate, three‐dimensional in vitro models for studying pathogenesis and drug screening [93, 179, 186–188]. While direct applications in disease prediction are still emerging, the “liquid biopsy” nature based on USCs‐EPs positions USCs as a potential source of biomarkers. USCs‐EPs may reflect the physiological or pathological state of the genitourinary tract and potentially systemic conditions, which positions them as promising sources of biomarkers for early disease diagnosis, dynamic monitoring, and prognostic assessment [187].

## 4. Conclusions

Stem cell therapy based on USCs, especially the USCs‐EPs, holds great potential for treating a diverse range of diseases in different organ systems. Even though further research is essential to comprehensively understand the potential of USCs and their derivatives and develop safer and more effective treatments, the preliminary findings are encouraging. USCs and USCs‐EPs might be promising tools with great application potential in the field of regenerative medicine.

## 5. Literature Search and Inclusion/Exclusion Criteria

A comprehensive literature search was conducted across three core biomedical databases: PubMed and Web of Science Core Collection, from their inception until May 2025. The search strategy employed a combination of keywords, including “urine‐derived stem cells”, “urinary stem cells,” and “USCs” paired with terms related to “paracrine”, “exosome,” and the specific organ systems (e.g., “bone” and “kidney”) outlined in the review scope. Studies were included if they were original research articles focusing on the paracrine mechanisms or related therapeutic applications of USCs. Exclusion criteria encompassed conference abstracts, studies not primarily investigating USCs or their paracrine effects, and non‐English publications. The final selection of literature was evaluated and synthesized to structure the narrative of this review.

**Nomenclature**
ADSCs: Adipose tissue‐derived stem cells
AKI: Acute kidney injury
APRIL: Aproliferation‐inducing ligand
ASCs: Adult stem cells
b‐FGF: Basic fibroblast growth factor
BAFF: B‐cell‐activating factor
BDNF: Brain‐derived neurotrophic factor
BMSCs: Bone marrow‐derived mesenchymal stem cells
BOO: Bladder outlet obstruction
CA: Cardiac arrest
ccRCC: Clear cell renal cell carcinoma
CPR: Cardiopulmonary resuscitation
DED: Diabetic ED
DN: Diabetic nephropathy
ECM: Extracellular matrix
ED: Erectile dysfunction
EphA 4: Eph receptor A4
EPs: Extracellular particles
ER: Endoplasmic reticulum
ESCs: Embryonic stem cells
EVs: Extracellular vesicles
Exos: Exosomes
GMCSF: Granulocyte‐macrophage colony‐stimulating factor
GROα: Recombinant human growth regulated protein
hPDLSCs: Human periodontal ligament stem cells
HDAC6: Histone deacetylase 6
HSCs: Hematopoietic stem cells
HUVECs: Human umbilical vein endothelial cells
IBD: Inflammatory bowel diseases
IDD: Intervertebral disc degeneration
iPSCs: Induced pluripotent stem cells
IRI: Ischemia‐reperfusion injury
IVD: Intervertebral disc
KOA: Knee osteoarthritis
KSFM: Keratinocyte‐serum‐free medium
MSCs: Mesenchymal stem cells
NOA: Nonobstructive azoospermia
NP: Nucleus pulposus
NRF1: Nuclear respiratory factor 1
NVEPs: Nonvesicular extracellular particles
PCL/GT: Polycaprolactone/gelatin
PD: Peyronie’s disease
PGE2: Prostaglandin E2
piGEL: Photo‐triggered imine crosslink hydrogel
PTEN: Phosphatase and tensin homolog deleted on chromosome ten10
RPE: Retinal pigment epithelial
RTT: Rett syndrome
SUI: Stress urinary incontinence
TA: Telomerase activity
UECM: USC‐derived ECM
USCs: Urine‐derived stem cell.

## Author Contributions

Dan Li conceptualized and wrote the first draft. Zhijuan Liang screened and collected the literature. Liping Wang, Yuanbin Chen, and Dongkai Sun contributed partial literature study. Ye Liang reviewed and finalized the manuscript.

## Funding

This study was financially supported by the Shinan District Science and Technology Program of Qingdao (Grant 2022‐2‐002‐YY) and the Shandong Provincial Natural Science Foundation (Grant ZR2021QH234).

## Disclosure

All authors read and approved the final manuscript.

## Conflicts of Interest

The authors declare no conflicts of interest.

## Data Availability

The data that support the findings of this study are available from the corresponding author upon reasonable request.
